# The Experience of Manual Wheelchair Training for People With Chronic and Progressive Conditions: Perspectives of Users and Trainers

**DOI:** 10.1111/hex.70342

**Published:** 2025-06-30

**Authors:** Kimberly Charlton, Carolyn Murray, Natasha Layton, Stacie Attrill

**Affiliations:** ^1^ School of Allied Health Science and Practice University of Adelaide Adelaide Australia; ^2^ Allied Health and Human Performance Academic Unit University of South Australia Adelaide Australia; ^3^ Rehabilitation, Ageing and Independent Living Research Centre Monash University Melbourne Australia

**Keywords:** chronic conditions, person‐centred training, progressive conditions, safe learning environments, wheelchair training

## Abstract

**Introduction:**

Global ageing and the rise of chronic and progressive health conditions that lead to mobility changes will see increased need for manual wheelchair (MWC) provision and training. Existing training guidelines and training programmes are frequently tailored towards younger users. There is a knowledge and practice gap regarding the needs of people with chronic or progressing conditions who require a wheelchair. To inform practice guidelines and training practices, this study sought the perspectives of both MWC users and trainers on their experience of MWC training.

**Methods:**

Using a qualitative descriptive approach, semi‐structured in‐depth interviews were conducted with 11 MWC trainers and 6 MWC users. Data from the two participant groups were inductively coded and thematically analysed using NVivo and concept mapping to synthesise the data into themes and sub‐themes.

**Results:**

Three main themes were identified: *guided support in wheelchair training* reflected the need for basic support when commencing wheelchair use, *person‐centred training* recognised the importance of tailoring training to individuals, their goals and contexts, and *creating safe and supportive environments* addressed how to foster acceptance of training through building a supportive training environment.

**Conclusion:**

Access to skilled MWC trainers is essential for MWC users commencing MWC use due to a chronic or progressive condition; however, the Australian healthcare system does not currently meet this need. There is a need to explore alternate models of service delivery, such as peer‐led training or upskilling of other key stakeholders, such as assistive technology suppliers. The creation of supportive environments and tailored training aligned with the abilities and goals of individual users must take precedence over resource‐driven or one‐size‐fits‐all approaches.

**Patient Contribution:**

During the development of semi‐structured interview guides, feedback was sought from an MWC user and MWC trainer to ensure the relevance and appropriateness of the questions and allow for the refinement of questions.

## 1 Introduction

Quality manual wheelchair (MWC) training can promote safety, independence and community participation for MWC users. This outcome can reduce health service use and reliance on informal carers [1]. Global demographic data suggests that 1% of the population requires a wheelchair to assist with mobility, and this requirement is predicted to grow with an increase in the older population and the prevalence of chronic and progressive conditions [2]. This trend in wheelchair use makes the provision of quality MWC training essential [2]. Wheelchair training has received some attention with recently published guidelines from the World Health Organization (WHO) [2] and guidelines prepared about upper limb preservation for wheelchair users [3]. While existing MWC training guidelines [2, 3] provide a valuable foundation, they are broad in nature and are not designed to meet the specific needs of different MWC users.

MWC users with chronic or progressive conditions are often older and may have reduced physical capacity (i.e., reduced strength, flexibility and endurance) [4], cognitive or sensory impairments [5], low confidence [4] and limited motivation to engage in training [6]. These factors highlight the need for training programmes that accommodate their functional abilities, slower learning pace, and lower confidence and motivation. Existing MWC training programmes, including WheelSeeU [7], Roulez Confiance (Rolling with confidence) [8], TEAM Wheels [9], EPIC Wheels [10] and the Wheelchair Skills Program [11], are of high quality but are largely designed and resourced for younger MWC users. This may leave MWC users who come to wheelchair use later in life due to a chronic or progressive health condition with insufficient or unsuitable training for their needs [12, 13, 14].

Existing literature provides insight into MWC training for older users that may also be relevant for those with chronic or progressive conditions. This includes recommendations about what content needs to be included in MWC training for this population and the types of physical environments that are valued [4, 15, 16]. Literature also suggests positive outcomes from group‐based [4, 17] and peer‐led training approaches [4, 17, 18]. While this attention to the needs of this population is positive, there remains a lack of understanding around how to support and adapt training to meet the needs of MWC users who may have reduced physical conditioning or cognitive and/or sensory changes that may impact how MWC training is taught. It is also unclear what supportive elements could be implemented to enhance confidence, address fear and anxiety and motivate users to engage in MWC training to facilitate safe MWC use.

Knowing more about the MWC training needs for users with progressive and chronic conditions, how MWC training is currently delivered, and the experiences of MWC users and trainers could lead to the development of recommendations for programmes that are tailored to their needs. This practice change may decrease injury risk for MWC users with progressive and chronic conditions, increase independence and social engagement, and reduce caregiver and health system strain. The following research question guided this exploration: ‘What are the perspectives of MWC users who commence wheelchair use due to a chronic or progressive condition, and the training providers who support them, regarding MWC training?’.

## 2 Methods

This study followed the Consolidated Criteria for Reporting Qualitative Research (COREQ) checklist [19]. Ethical approval for this study was obtained from the Human Research and Ethics Committee at the University of Adelaide (H‐2023‐290).

### 2.1 Study Design

A qualitative descriptive approach gathered insights from individuals who use wheelchairs due to chronic or progressive conditions, their caregivers and MWC training providers in relation to their experience of MWC training. The semi‐structured interview design allowed for open‐ended questioning with follow‐up probing questions and deep exploration of participants' thoughts, feelings and beliefs [20]. This approach kept interpretations of findings close to the data [21, 22].

### 2.2 Sampling and Recruitment

MWC users who commenced MWC use as an adult due to chronic or progressive conditions, caregivers, and providers of MWC training for this population were purposively sampled by the first author (K.C.). A chronic condition was defined as a health condition typically persisting over 3 months and a progressive health condition, which may worsen over time or require continuous management for its persistent effects [23]. Given that many people living with chronic and progressive conditions will acquire an MWC, due to affordability and availability, this study focused on MWC users and excluded people who solely used power wheelchairs. Individuals with spinal cord injury (SCI) were excluded from this study due to the substantial body of existing research focused specifically on MWC training for this population; however, trainers of individuals with SCI were included on the provision that they had experience in training individuals with chronic or progressive conditions. See Table 1 for full eligibility criteria. Recruitment occurred via social media platforms, Australian‐based assistive technology, aged and disability care organisations, Australian professional assistive technology bodies, allied health managers within healthcare services and snowball sampling, where participants referred other eligible individuals. The sample size was determined iteratively during data analysis [24].

**Table 1:** Eligibility criteria for participants.

Population	Inclusion criteria	Exclusion criteria
Providers of MWC training	Experience in the provision of manual wheelchair training to individuals who commence MWC use as an adult (over 18 years) due to chronic, progressive or age‐ related conditions Sufficient English language to participate in an interview	
Manual wheelchair users	Wheelchair users must have commenced MWC use as an adult (over 18 years) due to a chronic or progressive condition Cognitive and communication skills sufficient to communicate their opinions and experiences in an interview Any duration of experience in manual wheelchair use	Wheelchair users with a spinal cord injury, with no other underlying conditions/whose primary disability is a spinal cord injury Wheelchair users who solely use powered wheelchairs
Caregivers of MWC users	Caregivers of wheelchair users who commenced MWC use as an adult (over 18 years) due to a chronic or progressive condition	Caregivers of wheelchair users with a spinal cord injury, with no other underlying conditions/whose primary disability is a spinal cord injury

### 2.3 Data Collection

Participants provided demographic information via a Qualtrics survey (Qualtrics, Provo, Utah, the United States) and provided written or verbal consent before the interview commenced. The first author (K.C.) conducted individual semi‐structured interviews online via Zoom (Zoom Video Communications Inc.). Separate interview guides were developed for MWC trainers and MWC users based on findings from a recent scoping review on wheelchair training approaches [1]. The interview guide was piloted with an MWC trainer and an MWC user who were ineligible for the research and known to the research team. The guide was refined based on feedback. See Table 2 for interview guide questions. Interviews took place over a 11‐month period from January 2024 to November 2024 and lasted an average of 50.58 ± 7.93 min. Interviews were recorded and transcribed verbatim.

**Table 2:** Interview guide questions.

	MWC trainer	MWC user
General experience of training	Tell me about your experience with manual wheelchair training	Tell me about your experience with learning how to use a wheelchair. Can you tell me about when you first received your wheelchair?
Understanding of how existing MWC training supports users with chronic or progressive conditions	Tell me about any considerations you make when providing manual wheelchair training. What do you feel helps clients to become confident and independent in wheelchair use?	What helped you feel confident in using your wheelchair? And what helped you become more independent with it? What parts of your wheelchair training worked best in helping you learn to use your wheelchair?
Suggestions for improving MWC training	What advice would you give about how best to support people coming to wheelchair use as adults? In your opinion, what should wheelchair training look like? In your opinion, what are the main barriers to the delivery of high‐quality wheelchair training? In your opinion, what are the main facilitators in the delivery of high‐quality wheelchair training?	Were there things missing in your training that might have helped you? Can you tell me at least three things that you would like health services that provide training to know to support people who are transitioning to wheelchair use?
Concluding thoughts	Is there anything else you would like to tell us about wheelchair training that we haven't already covered in our interview?	Is there anything else you would like to tell us about wheelchair training that we haven't already covered in our interview?

### 2.4 Data Analysis

Braun and Clarke's six‐phase inductive reflexive thematic analysis was used to identify, analyse and report themes [24]. Reflexive thematic analysis was chosen due to it being a well‐established method for qualitative data analysis to identify patterns of meaning across data. Initially, data from MWC trainers and MWC users were analysed separately to ensure the voice of both groups was retained and centred across the data analysis and interpretation. The first author (K.C.) undertook multiple reviews of each transcript to understand the breadth and depth of the data, generating initial codes with descriptors and inductively sorting codes of similar meaning into categories using the NVivo qualitative software platform (QSR International Pty Ltd). The descriptions and initial categories were shared with and discussed among the research team, leading to further refinement. Findings from the MWC users and the MWC trainers were then combined using a concept mapping approach [25], which enabled identification of any overlapping, complementary and divergent data in the categories. A refined set of integrated themes was developed, incorporating both shared and unique elements across the two participant groups. These themes and sub‐themes were examined by the whole research team against the research question and were further refined to arrive at main themes with detailed memos documenting the rationale behind each theme. Table 3 shows an example of the relationship between a theme, sub‐theme, code and transcript data. Thick descriptions of the data and direct quotations were used to ensure the confirmability of the author's interpretation.

**Table 3:** Examples of sub‐themes, codes and raw transcription data for each theme.

Raw transcript data	Code	Sub‐theme
Theme 1: Guided support in wheelchair training		
‘*I regret not asking questions so much earlier. I'm in touch with other people in wheelchairs, and they will be like, can't you flip your front wheels up?’ (Delilah—MWC user)*	You don't always know what you might need to learn and what is important until it is too late.	‘You don't know what you don't know’
‘*I had the wrong team initially, when I was in hospital. I think if I'd had a different team behind me, I would have got up and been doing things a whole lot quicker.’ (Debbie—MWC user)*	Having trainers who know what they are doing is important	Getting the right person to help
‘*I wanted to basically know the best way to push with the least amount of energy. It took me a long way to figure out, and I'm still probably not even doing it right.’ (Chris—MWC user)*	Biomechanical education is important	Addressing the basics
Theme 2: Person‐centred training		
‘*My body is different to every other body. I've got really little arms, I've got contractures. There is no body like mine, and I would say the same for everyone else. So that needs to be considered when training is provided’ (Delilah—MWC user)*	Every MWC user is different in their body size and their abilities, so training needs to be tailored to an individual's needs.	Being sensitive to different needs
‘*It's important to ask questions like; where in your local community do you want get to? Where do you go and what do those environments look like? Are you a public transport user? It's about sort of, navigating what their needs are within their local community and lifestyle to shape how training will look.’ (Bianca—MWC Trainer)*	Considering the goals of a person and matching training to one's goals would be beneficial	Identifying person‐centred goals
‘*It would have been better to take me out into a shopping mall or plaza to give me an open environment with different surfaces and different things to manoeuvre around, because that potentially is where you're going to be using it [the wheelchair] the most.’ (Michelle—MWC user)*	Completing training in contextually relevant places is important	Contextualising training
Theme 3: Creating safe and supportive environments		
‘*Family and friend relationships make a big difference. If family and friends are very positive about wheelchair use, then that patient is more motivated. If family and friends are like not then that makes it harder for the patient to accept.’ (Francesca—MWC trainer)*	Having family and friends who are positive about wheelchair use and the benefits supports acceptance of MWC training and use	Building positive messaging
‘*Even if we're outdoors or something, I'll just sort of have a bit of chat about stuff and not just make it so focused on the wheelchair skills unless you know they've got some cognitive concerns, and you don't want them to be distracted. But yeah, I usually try and kind of keep it quite light.’ (Phoebe—MWC trainer)*	Having informal conversations not related to MWC training helps users relax and feel more comfortable engaging with training	Fostering a safety network
‘*It helped when he was behind me as I was giving it a go, because he making sure that I wasn't gonna tip. I had to tip bars on, but he was making sure I didn't do anything else.’ (Delilah—MWC user)*	Having anti‐tips or someone stand behind when completing wheelies can make the user feel safer when completing skills	Feeling safe
‘*We've got a number of sort of drills that we would send them home with like seeing how long you can get from one push or in a hallway see how few pushes you can do to get to the other end just to get them more confident with releasing the wheels and letting the wheelchair do some of the work for them in a more fun way.’ (Bianca—MWC trainer)*	You can use drills or gamify training to support an efficient propulsion style	Making training fun

### 2.5 Rigour

Credibility of findings was supported through use of a reflexive journal and peer debriefing before and during data gathering and analysis. Inclusion of a clear position statement acknowledges and supports the management of any subjectivity and assumptions that may have been held by the research team [26]. Supporting quotes from participants are used to illustrate themes. Data collection over an 11‐month period allowed for prolonged engagement and familiarity with the data and enabled iterative refinement of interview techniques. The first author maintained an audit trail of analytical decisions to allow backtracking and ensure the dependability of the final themes and confirmability of findings, which were strengthened through all members of the research team being involved in data analysis.

### 2.6 Author Positioning

The primary author is an occupational therapist and PhD candidate with experience in MWC training with people with chronic, progressive and ageing conditions within inpatient and community settings. This first‐hand experience provided insights into the challenges in providing and receiving high‐quality MWC training and has been the motivation for this study.

The co‐authors have occupational therapy and speech pathology backgrounds. All authors have experience in conducting qualitative research.

## 3 Results

### 3.1 Participants

Seventeen people from four states and one territory of Australia were recruited for this study. Demographic information is listed in Tables 4 and 5. Six MWC users (4F, 2M) participated in interviews. MWC users had an average age of 55.5 ± 14.74 years, and experience in MWC use ranged from 3 to over 15 years (average 6.25 ± 4.53). MWC use was related to lower limb amputations secondary to chronic conditions (*n* = 3), multiple contributing conditions (*n* = 2) and post‐polio syndrome (*n* = 1). Three users obtained a wheelchair while living in the community, and three within an inpatient setting, and all reported receiving minimal to no MWC training. All MWC users resided within Australia, with the majority living in urban areas (*n* = 5). MWC trainers (9F, 2M) participated in interviews, representing a range of training experiences, from emerging (0–5 years) to over 30 years. MWC trainers were most commonly occupational therapists (*n* = 6), followed by peer wheelchair trainers (*n* = 3), exercise physiologists (*n* = 1) and allied health assistants (*n* = 1). Training delivery experience was across government agencies (*n* = 8) and private practice settings (*n* = 8) and within both inpatient (*n* = 8) and community (*n* = 7) environments. The trainers predominantly provided their service to adult MWC users, including those with neurological conditions, amputations, orthopaedic injury and SCI. All participants were assigned pseudonyms, which are used to report the findings.

**Table 4:** Characteristics of MWC users.

Participant pseudonym	Gender	Age	Duration of MWC use	Reason for MWC use	Setting MWC was first obtained	Reported level of training provided	State MWC obtained
Delilah	Female	28	2–5	Multiple contributing conditions	Community	Minimal training from the AT provider	NSW—Major Urban
Lisa	Female	70	10–20	Post polio	Community	No training provided	ACT—Major Urban
Chris	Male	64	2–5	Multiple contributing conditions	Community	No training provided	QLD—Regional
Michelle	Female	52	5–10	Lower limb Amputee	Inpatient	No training provided	SA—Major Urban
Frank	Male	61	2–5	Lower limb Amputee	Inpatient	Minimal training provided by OT	SA—Major Urban
Debbie	Female	58	2–5	Lower limb Amputee	Inpatient	Minimal training provided by OT	SA—Major Urban

Abbreviations: ACT = Australian Capital Territory, AT = assistive technology, MWC = manual wheelchair, NSW = New South Wales, QLD = Queensland, OT = occupational therapist, SA = South Australia.

**Table 5:** Characteristics of MWC trainers.

Participant pseudonym	Gender	Years of MWC training experience	Professional background	Practice setting	Training location	MWC user demographics	State training provided within
Laura	Female	10–20	EP	Private	Community	Adults—various conditions, including neurological and SCI	Victoria—Major Urban Population
Hannah	Female	20–30	OT	Public and private	Inpatient and community	Adults—various, including degenerative conditions and SCI	Victoria—Major Urban Population
Vince	Male	10–20	Peer MWC Trainer	Private	Community	Adults—various, including MS, amputees, spina bifida and SCI	NSW and Victoria—Major Urban Population
Kane	Male	5–10	Peer MWC Trainer	Public and private	Community and inpatient	Adults—various, including MS and other degenerative conditions and SCI	Victoria—Major Urban Population
Melissa	Female	0–5	Peer MWC Trainer	Public and private	Community and inpatient	Children and adults—various, including amputees, spina bifida and SCI	Victoria—Major Urban Population
Veronica	Female	0–5	OT	Private	Community	Adults—various, including MS, general rehabilitation, cuada equina and SCI	South Australia—Major Urban Population
Francesca	Female	Over 30	AHA	Public	Inpatient— subacute	Adults—mostly stroke, other neurological conditions and amputees	South Australia—Major Urban Population
Libby	Female	10–20	OT	Public	Inpatient— subacute	Adults—stroke, ABI, other neurological conditions, amputees and SCI	South Australia—Major Urban Population
Bianca	Female	5–10	OT	Private and public	Inpatient—acute/subacute and community	Adults—various conditions including stroke, MS, other neurological conditions, amputees and orthopaedics	South Australia—Major Urban Population and the UK
Pheobe	Female	5–10	OT	Private and public	Inpatient— acute/subacute	Adults—various conditions including neurological conditions, amputees, bariatric clients, orthopaedics and SCI	South Australia—Major Urban Population and the UK
Kristen	Female	10–20	OT	Public	Inpatient— subacute	Adults—various conditions including neurological conditions, amputees, orthopaedics, Guillain‐Barré and SCI	South Australia—Major Urban Population and the UK

Abbreviations: ABI = acquired brain injury, AHA = allied health assistant, EP = exercise physiologist, MS = multiple sclerosis, MWC = manual wheelchair, NSW = New South Wales, OT = occupational therapist, SCI = spinal cord injury, UK = United Kingdom.

**Figure 1:**
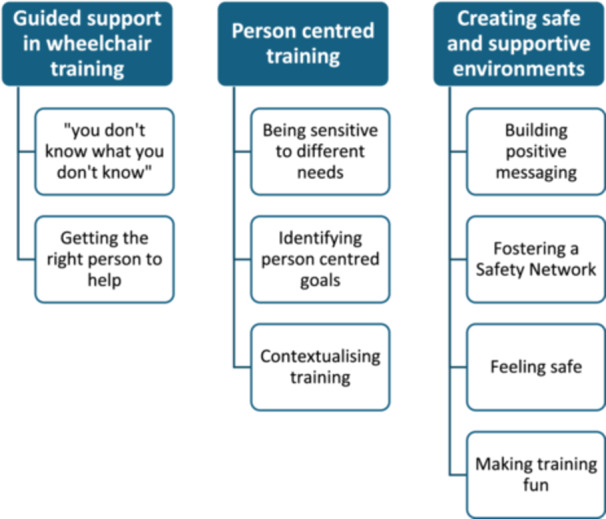
Overview of themes and sub‐themes.

Experiences of wheelchair training from the perspective of users who began MWC use due to a chronic or progressive condition and of MWC trainers are presented across three themes and nine sub‐themes. Figure 1 provides an overview of themes and sub‐themes, which are explored below.

### 3.2 Theme 1: Guided Support in Wheelchair Training

The data provided insights into MWC users' experiences of receiving limited guidance or training in MWC use and the consequences of this, as well as suggestions on who should support training and what training should encompass.

#### 3.2.1 ‘You Don't Know What You Don't Know’

MWC users reported that minimal education on wheelchair use often led to frustration and unsafe techniques, resulting in injuries or an avoidance of MWC use and reliance on walking and its associated risks.

‘I knew nothing. Which actually is why the incident [fall from MWC] happened, because I didn't know what I was doing.’

Michelle—MWC user

‘It got to the stage that I didn't feel safe, and I didn't feel comfortable [in the MWC]. So I then forced myself when I went out in the public to use my walking sticks. That put me at an increased risk of falls and overheating, and overexertion will bring on seizures.’

Chris—MWC user

‘It would have been good to learn basically the best way to push with the least amount of energy … it took me a long time to figure it out, and I'm still probably not even doing it’.

Delilah—MWC User

For others, a lack of MWC training not only led to greater dependency, reduced confidence and restricted community participation but also diminished their sense of agency.

‘I couldn't push myself up any slope…. So I would have support workers, drop me to work, push me down the hill into work, and then they would come at 5pm and do the same thing.’

Delilah—MWC user

#### 3.2.2 Getting the Right Person to Help

Three MWC users who commenced using a MWC in the community noted that wheelchairs were often acquired through assistive technology suppliers or garage sales, without professional support. The three MWC users who acquired a wheelchair through a hospital setting were provided with their wheelchairs by a professional, but this did not always result in users being able to use their wheelchairs. Many found their first MWC experience daunting, highlighting the need for skilled, supportive and knowledgeable trainers to provide clear instruction and instil confidence.

‘We need to have the training and the confidence to present something because otherwise if we go in half hearted, then we sound like we don't know what we're talking about, then that doesn't give clients confidence in us.’

Veronica—OT

MWC users expressed challenges in accessing quality trainers due to funding and resource constraints within hospitals and the community, and trainers explained that competing work demands reduced face‐to‐face training time with clients.

‘I went for about 6 months, not being able to get one [an OT] because there just wasn't anybody free to help.’

Lisa—MWC user

Opinions differed on who should provide MWC training. Peer‐led trainers and some MWC users and trainers, who were health professionals, felt peer training was important in supporting engagement in training but peer trainers reported ‘ring fencing’ around who could provide MWC training. Other health professionals justified their involvement.

‘Lots of people have told me this. They say you've been my inspiration. I saw you do this, and I thought, Well, if she can do it, I'll give it a go, too.’

Melissa—Peer MWC Trainer

‘I think the OTs [occupational therapists] should be the ones involved in training, because we do home visits and function, retraining all of that.’

Kristen—OT

### 3.3 Theme 2: Person‐Centred Training

MWC users and trainers recognised the diverse abilities, goals and stages of readiness of those requiring wheelchairs. This theme emphasises the importance of tailoring training to individuals, creating person‐centred goals and delivering contextually appropriate training.

#### 3.3.1 Being Sensitive to Different Needs

MWC trainers agreed that training should be tailored to users' abilities and readiness, while remaining flexible to accommodate daily changes in mood or physical condition. However, MWC users noted that training was often predetermined based on checklists that did not consider users' abilities, goals or personal circumstances.

‘They had a set of things they got me to do and then that was it’

Debbie—MWC user

Understanding a user's physical abilities (i.e., balance, endurance, upper limb strength, sensory abilities and shoulder pathologies), cognitive abilities (i.e., safety awareness and comprehension), psychological readiness (i.e., emotional readiness, injury acceptance and confidence) and psychosocial and/or mental health comorbidities were identified as being important for guiding the training process.

‘You've got to consider things like, do they even have upper limb strength to push themselves?’

Bianca—OT

‘Sometimes people aren't ready yet, like emotionally for a wheelchair. A lot of people have trouble accepting their injury and adjusting to their injury.’

Libby—OT

To support ageing individuals with chronic or progressive conditions, MWC trainers highlighted the importance of adjusting session length, frequency and incorporating rest breaks and repetition. They recommended breaking learning into manageable chunks and building skills gradually to support confidence. Mastery of skills incrementally was suggested to support sustained motivation.

‘We use the 4 C's. You have to show correct technique, consistently, you must appear confident, feel comfortable doing it, and you have to show that skill at least 4 times before we move on.’

Melissa—Peer MWC Trainer

MWC trainers suggested including verbal and practical demonstration and diverse feedback methods, including verbal cues, visual aids (e.g., mirrors or video), tactile support (e.g., hand over hand) or kinaesthetic input to support users' perceptual, visual and comprehension abilities.

‘So particularly with the older population … giving that practical demonstration, visually showing them makes a lot of difference.’

Phoebe—OT

#### 3.3.2 Identifying Person‐Centred Goals

Participants valued the role of person‐centred goals in supporting motivation, engagement and training relevancy. Assessment tools were sometimes used to guide the goal‐setting discussion.

‘It tends to highlight the things that are possible in a chair and determine whether or not that's a goal for that person … so we use that assessment (WST‐Q) to guide the conversation about what their goals are’.

Laura—EP

Exploring an MWC user's environment and desired activities also informs goal setting. MWC users identified that goals centred around their community participation, including shopping or leisure‐based tasks, made training more engaging and less monotonous. Trainers emphasised the value of starting with small achievable goals to build momentum and trust and to break down larger challenges into achievable steps.

‘If you get that easy win you've then got them the whole way, because once they've seen the value in it, they'll then go right, Okay.’

Vince—Peer MWC Trainer

Despite the emphasis on goals led by MWC users, all participants acknowledged that training is often shaped by the setting. In acute care, the focus is typically on ensuring safe hospital discharge, rather than addressing broader, person‐centred goals. This practice can result in training that does not progress beyond basic skills or extend to outdoor or advanced skills, even when MWC users and trainers recognise the importance of such skills.

#### 3.3.3 Contextualising Training

While hospital‐based MWC training provides a safe, controlled environment for learning, MWC users and trainers agreed that this does not prepare users for the complexities of everyday navigation or attend to their goals. Training within home and community settings allows for the identification of challenges, fosters problem‐solving and makes training more meaningful, engaging and relevant.

‘The initial training in a hospital if you are just in a safe environment. You're only ever gonna expect it to be safe. And life's not like that.’

Frank—MWC user

Despite this recognised importance, MWC trainers explained that resource limitations often restricted the provision of these training opportunities, leaving users reliant on others to navigate unpredictable environments. To address this, some hospital‐based trainers utilised purpose‐built facilities or nearby community areas to teach mobility skills, but MWC users noted that these settings did not always reflect real‐world challenges.

### 3.4 Theme 3: Creating Safe and Supportive Environments

Engaging in MWC training can be challenging, making it essential to create a safe, supportive environment. This theme discusses fostering acceptance of training through building a supportive network, creating a sense of safety and making training fun.

#### 3.4.1 Building Positive Messaging

For some people, a wheelchair can be seen as a sign of defeat, injury, disability and trauma and adjusting to MWC use can be challenging, especially in acute settings where other priorities often take precedence. MWC trainers expressed that many individuals experienced shame, reluctance or a pessimistic attitude towards MWC use and frequently placed a higher value on regaining their walking ability, rather than learning to use a wheelchair.

‘Part of that emotional journey is the shame that you feel, the embarrassment that you feel to start off with. When you come to this later in life, the sense of a loss of freedom.’

Chris—MWC user

‘A lot of patients are very resistant to being in a chair, and they're like, I won't be needing this, because I will be walking.’

Francesca—AHA

These attitudes can sometimes be reinforced by allied health professionals who prioritise walking training over MWC training.

‘The physiotherapist would be working on them every day to get them to the point that when they left the hospital they could walk. Maybe 5 meters. Being able to walk 5 meters, is not functional’

Vince—Peer MWC Trainer

MWC users and trainers valued psychological support in fostering acceptance of MWC training, suggesting that with the right support and attitudes from health professionals, peers and family/friends, a wheelchair can be reframed as a tool for independence.

‘Sometimes with rehab, you need to have the family on side … because the people that you trust are often the ones that will get you going.’

Debbie—MWC user

MWC trainers noted that MWC users are more likely to engage in training once they realise its benefits. They suggest highlighting these advantages in a personalised way, tailored to what matters most to each user.

‘Getting back out to smoke and not being able to do it in hospital grounds. That really motivates.’

Bianca—OT

#### 3.4.2 Fostering a Safety Network

MWC trainers saw the need to engage in informal conversations to foster supportive, emotionally safe learning environments and positive trainee–trainer relationships. Hierarchical dynamics between allied health professionals and MWC users were seen to create power imbalances, reduce a user's confidence and discourage open communication.

Peer trainers felt group training created safety and a community for MWC users, through providing opportunity to share practical strategies to overcome challenges. MWC users appreciated how group training shifted focus away from themselves, making the learning experience less daunting.

‘Being surrounded by people that are learning the same skills, I think that's a really big support in terms of their learning.’

Melissa—Peer MWC Trainer

Having family/friends present during training was seen to build confidence, reduce social isolation and provide encouragement for users to attempt challenging skills and enable practice beyond formal training sessions. Carer education was identified as being pivotal for the safety of an MWC user; however, caregiver involvement in training was often incidental rather than planned.

‘It would make them feel more comfortable if they've got a family member there.’

Libby—OT

Some MWC users found safety in learning at their own pace through self‐directed observation of others, either in person or online. However, one MWC user (Michelle) cautioned against replicating online content without due consideration, as some skills could be dangerous for users with reduced physical capacities, including those who are ageing with chronic or progressive conditions.

‘There is quite a lot out there that makes it seem like everyone can do that [go up and down stairs] with a wheelchair. But, probably only 10% can actually do it in a wheelchair’

Michelle—MWC user

#### 3.4.3 Feeling Safe

Participants explained the need to introduce safety features on MWCs, like brakes and anti‐tip bars, and to teach risk mitigation strategies. These were crucial inclusions for building trainee confidence. The use of spotter straps and completing controlled practice of skills also contributed to a user feeling confident and safe.

‘He was behind me as I was giving it a go, making sure that I wasn't gonna tip.’

Delilah—MWC User

Indoor training was widely regarded by participants as an ideal starting point for MWC training, offering a controlled space for users, free from the unpredictability of outdoor settings. Trainers could then gradually introduce challenges which may replicate outdoor scenarios to match an MWC user's performance and confidence progression.

‘I found teaching those skills in a closed environment much easier and then transitioning to the community.’

Laura—EP

Environmental risk assessments further contributed to creating safe learning spaces by ensuring training locations were suitable and safe. Educating MWC users about environmental risks empowered them to navigate their surroundings with heightened awareness and confidence to support safe problem‐solving.

‘The average person who goes into a wheelchair has no idea.’

Chris—MWC User

#### 3.4.4 Making Training Fun

The inclusion of fun, personalised MWC training was suggested by MWC users and trainers. They rationalised that by making MWC training an enjoyable experience, users feel less burdened by the learning process and more willing to engage in training opportunities.

‘Make it fun. If you're training with OTs, get them to have a kit bag of things that make training fun.’

Debbie—MWC user

‘Generally having that license to have a bit of fun helps to break the ice for everyone. I think the wheelchair becomes something different. It doesn't become a symbol of defeat and injury and disability and trauma.’

Vince—MWC Trainer

Suggestions for building fun included: incorporating music into training, icebreaker activities and gamifying training to support efficient propulsion style. Incorporating social elements like morning tea or informal networking before or after training was also suggested by Vince (Peer MWC trainer) to encourage relaxed ‘car park’ or ‘sideways’ conversations, where people feel comfortable to ask questions and share experiences.

## 4 Discussion

This study gathered the perspectives of MWC users with chronic or progressive conditions and MWC trainers on their experiences of training, to explore ways to support the physical needs, confidence and engagement of this population. Three key themes emerged from the experiences of MWC training in both participant groups. These included the need for guidance from knowledgeable trainers, the value of person‐centred approaches and the need for safe, supportive environments for learning.

Both MWC users and trainers in this study identified peer‐led training as being valuable for building confidence and motivation, due to the relatable insights and support that peers can provide. This finding is consistent with other peer‐led MWC training programmes, which suggest that peer‐led training is effective for building trust, motivation, engagement, self‐efficacy and skill acquisition [8, 9, 17, 27, 28, 29]. As such, expanding existing peer‐led initiatives in Australia, such as *Skills for Independence* [30], could enhance both accessibility and effectiveness of training delivery for this population of MWC users.

While peer‐led training may be one mechanism to support the self‐efficacy and motivation for MWC training in those with chronic or progressive conditions, a person‐centred approach was also recognised in this study to be essential for maintaining an MWC user's motivation and ensuring that physical and cognitive needs are met. Consistent with other MWC training research, users are more likely to remain engaged and invested when training is tailored to their goals [4, 6, 8, 9, 10, 27, 31, 32], provided within environments that are important and contextually appropriate for the user [17, 27, 33, 34, 35], and flexible and adaptable to meet the content and pace of an individual user [7, 18, 28, 29, 36]. This approach aligns with self‐determination theory [37], which highlights that when MWC users feel in control and find that training is relevant to their learning, there is a greater sense of autonomy and empowerment. This outcome will foster intrinsic motivation, thus contributing to engagement in training and improved safety, confidence and independence.

Accessibility to MWC training also came up as an issue in the research. Accessibility may be enhanced through upskilling those around the MWC user such as allied health assistants, assistive technology providers and carers through opportunities, such as WHO's online Training in Assistive products (TAP) [38], International Society of Wheelchair Professionals (ISWP) training programmes [39] and WHO wheelchair service provision training packages [40]. Incorporating caregivers into training was endorsed by the WHO's Wheelchair Provision Guidelines [2] and reinforced by existing MWC training literature [17, 34]. Stronger relations and referral pathways between primary healthcare providers (i.e., general practitioners), assistive technology retailers, hospital staff and community‐based MWC training providers could also improve access to MWC training. This approach aligns with contemporary recommendations from the WHO/UNICEF [41] to develop capability and capacity across health and social sectors. These are feasible suggestions for increasing access to MWC training and therefore improving efficient MWC propulsion.

Creating a safe, positive and supportive learning environment was important to enhance the MWC user's self‐efficacy. This learning includes reframing MWC use as a path to independence rather than associating it with a loss of independence. Inclusion of caregivers in training is one way to enhance safety, reinforce positive attitudes and sustain motivation. Group training is also recognised in the literature as creating positive learning environments through a sense of community [4, 7, 8, 27]. Group environments allow users to observe others succeeding, which helps them believe in their own ability to achieve similar outcomes. This relationship reflects Bandura's Social Cognitive Theory [42], which emphasises the role of observational learning, social reinforcement and self‐belief in shaping motivation.

## 5 Strengths and Limitations

This study captured an Australian perspective of MWC training across a range of healthcare settings, providing a well‐rounded understanding of current practices and challenges in MWC training delivery. Whilst we intended to recruit MWC users who had accessed training, all MWC users interviewed expressed receiving little to no MWC training, highlighting systemic barriers within the Australian healthcare system. These challenges may stem from MWC training remaining a hospital‐centric service delivery model, where training is led primarily by health professionals working in under‐resourced environments with a strong focus on discharge planning. Access may also be limited by fragmented community‐based supports, which vary depending on locality, age and individuals' financial resources. As the contexts of wheelchair use and training differ across countries, the findings of this study may not translate beyond Australia; however, a lack of guidance and education in MWC use has been reported in other high‐income countries, including Canada [12, 14, 43] and the United Kingdom [13]. Therefore, suggestions and insights generated through this study may have implications for MWC training internationally.

While the research gathered insights from experienced MWC trainers, working across diverse healthcare settings, most trainers had more experience in working with younger MWC users (i.e., SCI) than those with chronic and progressive conditions. Additionally, only six MWC users were interviewed in this study, and those who participated were eager to engage in this study as a mechanism to support and advocate for a change to the delivery of MWC training. This study, therefore, did not capture the perspectives of MWC users who had received comprehensive MWC training, or MWC users who were less engaged, nor did it capture caregiver perspectives. These results may therefore not fully reflect the diversity of MWC training experiences.

## 6 Conclusion

This study highlights gaps in MWC training for individuals with chronic or progressive conditions, emphasising that the current Australian healthcare system does not meet the needs of these users. There is a need to explore alternate models of service delivery, such as peer‐led training or upskilling of people around the MWC user to meet this need. A person‐centred approach that prioritises goal‐directed training, tailored to the physical and cognitive needs of the user and delivered within contextually relevant environments, is critical for fostering engagement and meaningful skill development. When considering the delivery of MWC training, leveraging principles from social cognitive theory and self‐determination theory could be used to provide enhanced motivation and self‐efficacy to support positive outcomes for MWC users.

## Author Contributions

**Kimberly Charlton:** conceptualisation, investigation, methodology, formal analysis, writing – original draft, writing – review and editing. **Carolyn Murray:** conceptualisation, methodology, supervision, formal analysis, writing – review and editing. **Natasha Layton:** conceptualisation, methodology, supervision, formal analysis, writing – review and editing. **Stacie Attrill:** conceptualisation, methodology, supervision, formal analysis, writing – review and editing.

## Conflicts of Interest

The authors declare no conflicts of interest.

## Data Availability

The data that support the findings of this study are available upon request from the corresponding author. The data are not publicly available due to privacy or ethical restrictions.
